# Expert Status and Performance

**DOI:** 10.1371/journal.pone.0022998

**Published:** 2011-07-29

**Authors:** Mark A. Burgman, Marissa McBride, Raquel Ashton, Andrew Speirs-Bridge, Louisa Flander, Bonnie Wintle, Fiona Fidler, Libby Rumpff, Charles Twardy

**Affiliations:** 1 Australian Centre of Excellence for Risk Analysis, School of Botany, University of Melbourne, Parkville, Australia; 2 Melbourne School of Population Health, University of Melbourne, Parkville, Australia; 3 Commonwealth Centre for Applied Environmental Decision Analysis, School of Botany, University of Melbourne, Parkville, Australia; 4 C4I Center, George Mason University, Fairfax, Virginia, United States of America; Hungarian Academy of Sciences, Hungary

## Abstract

Expert judgements are essential when time and resources are stretched or we face novel dilemmas requiring fast solutions. Good advice can save lives and large sums of money. Typically, experts are defined by their qualifications, track record and experience [1], [2]. The *social expectation hypothesis* argues that more highly regarded and more experienced experts will give better advice. We asked experts to predict how they will perform, and how their peers will perform, on sets of questions. The results indicate that the way experts regard each other is consistent, but unfortunately, ranks are a poor guide to actual performance. Expert advice will be more accurate if technical decisions routinely use broadly-defined expert groups, structured question protocols and feedback.

## Introduction

Expert judgments are attractive when time and resources are stretched, and are essential where data are inadequate, circumstances are unique, or extrapolations are required for novel, future and uncertain situations. Typically, experts are defined by their qualifications, track record and experience [1], [2]. People believe that experts have privileged access to knowledge and use it effectively [3], [4]. Society generally, and experts in particular, expect that more experienced, better-qualified and more highly regarded experts perform better when estimating facts within their domain of expertise. We call this the social expectation hypothesis.

Many scientific disciplines including ecology, health and engineering routinely depend on expert scientific judgments [5], [6], [7]. The growing recognition of expert judgment is underlined by recent innovations in methods for obtaining and combining expert estimates [8], [9]. Social expectations of expert performance appear to be well founded. Expert estimates of facts are generally better than lay estimates, within the expert's area of expertise [10], [11], [12]. Judgments have been shown to improve with experience in domains as diverse as physics and weather forecasting [12], [13].

However, these examples are from contexts in which feedback is direct, personal, unambiguous and immediate, from which the experts learn. For example, the judgments of weather forecasters are attributed to the person making them, and their predictions are verified by events in the following days. Extrapolations about expert performance in relation to experience may be misleading if applied to all expert judgments. For example, for either relatively complex or relatively simple problems, experts don't outperform novices as they do for intermediate problems [14], although what qualifies as complex is context specific and may be difficult to determine *a priori*. Experts may perform poorly when knowledge environments are lenient, feedback is weak, or experts are required to adapt to new situations [12], [15]. In addition, experts (and lay people) are sensitive to a range of psychological idiosyncrasies, subjective biases, values and conflicts of interest [6], [11], [16], [17].

Previous studies have demonstrated that better-calibrated and more accurate judgments can result from systems that provide feedback about performance, encourage experts to think about their estimates and aggregate individual estimates [12], [18], [19], [20], [21], [22]. However, no study previously has related actual performance, qualifications, track record and experience to both self-assessments and peer expectations of performance.

The purpose of this research is to test the *social expectation hypothesis* by examining the relationship between perception of expertise and the actual performance of scientific experts on test questions about facts. As is explained below, the hypothesis predicts strong correlations between, i) self and peer assessments, ii) peer assessments and experience, publications and qualifications, and iii) peer ranks and actual performance. We tested the hypothesis by comparing self-assessment with peer assessment of expected performance in groups of experienced life-science professionals, and by examining the relationships between attributes of expertise and expert estimates of relevant facts. This information was gathered in six structured elicitation exercises [1], [18] involving 123 experts (124 participated and 123 provided responses) from life science fields including medicine, epidemiology, veterinary science, ecology and conservation biology.

## Methods

We developed a structured procedure for questioning experts based on the methods of Cooke [18], [19] and Speirs-Bridge et al. [23]. This approach is designed to ameliorate the effect of dominant individuals in a group and mitigate some of the most pervasive and influential sources of bias including anchoring, dominance and overconfidence [11], [12], [16], [17].

### Elicitation protocol

Questions from the knowledge domain of groups of experts were used to test the performance of people along a continuum of expertise. Experts were selected by the people organizing the workshops in each case, and were included because of their relevant technical experience and training for the problem under consideration. We obtained approval for this project from the University of Melbourne's Ethics Committee and written approval of informed consent from all participants. Experts were provided with five to ten factual questions relevant to their expertise. To handle variation in response scales for each question, we first range-coded the estimates by each expert for each question. We then rescaled the answers to ameliorate the influence of outliers (see below). Standardization was important to ensure that each question contributed more or less equally to overall assessments of group performance. The rescaling described below provided a measure of deviation of each expert's estimate from the true value that was not dominated by a single prediction far from the observed value. The procedure was as follows:

1. Participants in a facilitated workshop situation completed a survey that documented their training, experience in the relevant area, professional role, memberships and publications.
2. Participants provided one another with a brief verbal summary of their training, professional experience and current role. Participants were then asked to privately rank themselves and the other participants on an 11-point scale (0 =  ‘no expertise’, 5 =  ‘moderate expertise’, 10 =  ‘highly expert’), representing how well they expected themselves and others to perform on test questions relevant to the topic of the meeting. We used the average of the values for each expert assigned by other experts as a measure of status.
3. A set of ‘fair’ questions [18] was developed from pre-publication experimental results, regulatory data bases and other relevant sources. The reasonableness of the questions was confirmed by one of the participants, usually the person who arranged the meeting.
4. The questions asked for quantities, natural frequencies [24] and probabilities using a four step procedure [23] in the following format:
  - Realistically, what is the lowest the value could be?
  - What is the highest the value could be?
  - What is your best guess (the most likely value)?
  - How confident are you that the interval you provided contains the truth (give a value between 50% and 100%)?
5. Participants completed each question by hand on paper questionnaires, resulting in an initial, private estimate of each fact.
6. The full set of individual estimates was displayed to the group visually (transformed to be 80% credible bounds around the best guess) using software developed for this process. The rescaling to an 80% interval assumed normal, lognormal or triangular distributions, depending on the context and data. Alternative assumptions were not critical as they did not make much difference to the visual display. Differences were discussed, question by question, with the aid of the facilitator. Participants were given the opportunity to resolve the meanings of words, specify context, and introduce and cross-examine relevant information.
7. Participants then provided a second, final private answer to each question.

### Expert workshops

The procedure was applied to groups ranging in size from 13 to 25 professionals, meeting to discuss health and biosecurity issues, to participate in training, and to estimate facts for risk assessments (Table 1). The participants represented disciplines including medicine, epidemiology, animal and plant health, ecology and conservation biology. Expertise in each group ranged from highly credentialed people with many years experience to relative novices with modest relevant training.

**Table 1:** Characteristics of the expert groups.

Workshop	Discipline	Numbe of experts	Location/Date	Range of years of relevant experience	Range of qualifications	Range of number of publications
1	Animal and plant biosecurity and quarantine	21	Melbourne, February 2010	0–37	BSc, BASc, BVSc, BCom, Grad. Dip., MSc, PhD	0–113
2	Animal and plant biosecurity	24	Christchurch, March 2010	0–39	BSc, MSc, MBA, MCom, PhD	0–270
3	Ecology, frog biology	13	Melbourne, March, 2010	0–42	BA, BSc, BSc (Hons), M Env Studies, PhD	0–45
4	Public health, medicine	25	Canberra, May 2010	0–45	BEng, BSc, BEcon, LLB, MBBS, Grad. Dip., MA, MSc, MBA, PhD	0–220
5	Risk analysis, biosecurity	20	Sydney, September 2010	0–40	BEng, BSc, BVSc, MBBS, Grad. Dip., MA, MBA, PhD	0–225
6	Weed ecology	20	Melbourne, December 2010	0–50	BSc, MSc, PhD	1–220

We applied appropriate questions to professional groups in six workshops, between February and December 2010. Workshops lasted from a few hours to two days. A trained facilitator oversaw the elicitation protocol and group discussion at each workshop. The workshops were generally in the areas of animal, plant and human health, and all were run in Australia or New Zealand. No expert participated in more than one workshop. In some workshops, most participants knew one another professionally. In others, most participants were unfamiliar with one another, so in these cases peer ranking was based on a brief verbal summary. The qualitative outcomes described below were not affected by these differences.

The preliminary information gathering and the first round of questions (steps 2 to 5 above) were conducted in the first one or two hours of the workshop. The information was compiled and the intervals standardized using software developed to support the procedure. The discussions were conducted and the second (final) round of answers were gathered in the last hour of the workshop. The questions varied between workshops to suit the particular skills of the participants (see Supporting Information S1). There were some variations in the details of procedures between workshops; specifically, in workshops 2 and 3, half of the participants saw the estimates of other participants but did not discuss differences before making a second judgment. Excluding people who did not talk with one another made no important difference to the results reported below.

We scored expertise using three simple measures; years of experience, number of relevant professional publications, and rank of highest relevant qualification (no tertiary qualifications = 0, any non-relevant degree = 1, relevant non-graduate qualification = 2, relevant degree = 3, relevant four-year degree = 4, relevant five year degree (veterinary science, medicine) = 5, relevant masters  =  6, relevant PhD = 7). Experts recorded their years of relevant experience and the number of professional publications they had authored or coauthored including peer reviewed papers, books, book chapters, and official (grey literature) reports.

### Evaluation of responses

Accuracy and bias may be measured by the correspondence between judgments and facts [18], [25], [26]. Calibration reflects how well the bounds specified by an expert accurately reflect uncertainty [23]. Evaluation of expert performance is most commonly done using scoring rules [27], [28], [29], which are functions that convert information about the expert's prediction and the true realization of the event to a reward [28]. Scoring rules differ primarily by the degree to which they reward and penalize various types of correspondence, bias and information. Different approaches are appropriate depending on whether the purpose is evaluation of performance or aggregation of results [19], [30], [31], [32]. In this case, the aim is to assess the accuracy of each expert's best guess, and we applied calculations to create a standardized distance from the truth for each person, averaged over all questions they answered [32].

Scoring rules for point estimates assess performance in terms of the average distance between the predicted and observed value across a set of predictions, standardized to account for differences in question scales [32]. In assessing performance, we were particularly interested in a measure that allowed performance on each question to contribute equally. The range of question types made it difficult for any one standardization method to cope with the full range of responses. To handle variation in response scales for each question, we first range-coded the estimates by each expert for each question. That is, we expressed each answer as,
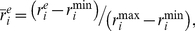
(1)where 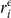 is the estimate from expert *e* for question *i*, 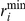 is the group minimum for question *i* and 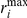 is the group maximum of the experts' estimates for the question (including the true value). The standardization ensured that each question contributed more or less equally to overall assessments of group performance. We then rescaled the answers, expressing each as the average log-ratio error (*ALRE*),
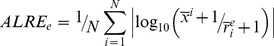
(2)where *N* is the number of questions given to a workshop group, *r* is the standardized prediction and *x* is the standardized observed (true) value. The log-ratio provides a measure of deviation of an expert's estimate from the true value that is not dominated by a single prediction far from the observed value. A prediction that is 10-fold greater than the observed value weighs as heavily as a prediction that is one-tenth the observed value. Smaller *ALRE* scores indicate more accurate responses and a perfect *ALRE* score would be 0. Using the standardized responses, for any given question the log ratio scores have a maximum possible range of 0.31 ( = log(2)) which occurs when the true answer coincides with either the group minimum or group maximum.

Commonly applied methods for estimating accuracy include the Mean Absolute Percentage Error (MAPE), which gives the average percentage difference between the prediction and observed value, and Root Mean Square Percentage Error (RMAPE), which is the square root of the MAPE. However, MAPE and RMAPE are strongly affected by one or a few very divergent responses.

## Results

Expectations of performance generally correlated with the normal measures of expert status, namely, years of experience, number of publications and academic qualifications (Table 2). Weak correlations between qualifications and peer assessments in Workshops 2 and 3 arose because all participants had relevant postgraduate qualifications, so that participants could not clearly rank expert status on this basis. The otherwise generally strong positive correlations (*r*>0.5) between these factors underline the structure of the *social expectation hypothesis*. That is, society and experts assume that experts have privileged access to knowledge gained through specialist training and relevant experience and that they are able to access and use this knowledge effectively. Experience, track record and qualifications are assumed to be reliable guides to this expertise.

**Table 2:** Pearson correlations between peer assessments of expert performance and measures of expertise, and between self-assessed expertise and peer assessments of expertise.

Workshop	Peer assessment versus years of experience	Peer assessment versus number of publications	Peer assessment versus qualifications	Self assessment versus peer assessment
1	**0.550 (n = 20)**	0.348 (n = 21)	**0.556 (n = 21)**	**0.675 (n = 21)**
2	**0.487 (n = 19)**	**0.587 (n = 21)**	0.064 (n = 20)	**0.684 (n = 24)**
3	**0.514 (n = 13)**	−0.123 (n = 13)	0.019 (n = 13)	**0.853 (n = 13)**
4	**0.591 (n = 25)**	**0.500 (n = 25)**	**0.489 (n = 25)**	**0.899 (n = 25)**
5	**0.836 (n = 20)**	0.309 (n = 17)	0.203 (n = 20)	**0.853 (n = 20)**
6	**0.620 (n = 14)**	0.289 (n = 14)	0.074 (n = 14)	**0.944 (n = 14)**

Statistically significant correlations (at α = 0.05, two-tailed) are in bold face.

**Figure 1:**
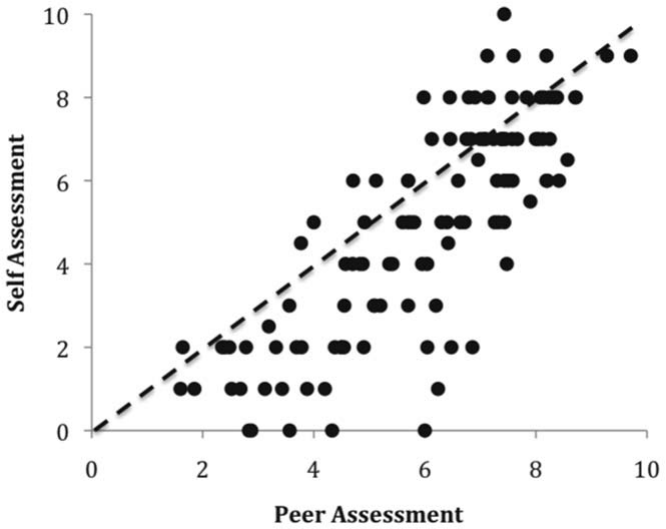
Self assessment versus peer assessment of expertise. Data from all workshops (overall correlation, *r* = 0.85). Peer assessment is the average of the scores on the 11-point scale provided by each person's peers on the day of the workshop. The strong relationship was consistent across the five groups, where the correlations ranged from 0.67 to 0.94 (Table 3). The dashed line is parity (where self assessment and peer assessment are equal).

The community of experts sampled in this study held the strong belief that more experienced and better-credentialed experts would perform better. The pervasiveness of this belief is reflected particularly in the correspondence between self-assessments and peer assessments of performance where correlations range from 0.68 to 0.94 (Table 2). Figure 1 combines the six workshops and shows this relationship as a scatter plot. People tended to rank themselves in a very similar fashion as their peers ranked them. Perhaps most surprisingly, people established consistent and highly correlated expectations of performance of their peers that accorded closely with the self-evaluations, even in workshops in which the participants were mostly unfamiliar with one another. These assessments were based on a very brief introduction (taking about one minute), in which they outlined their experience, track record and qualifications (Workshops 1 and 5).

**Table 3:** Correlations between peer assessments of expertise and the accuracy of predictions.

Workshop, number of participants, number of questions	Peer assessment versus prediction accuracy – first round	Peer assessment versus prediction accuracy – second round
1, n = 21, 10 questions	−0.391	0.119
2, n = 24, 10 questions	0.215	−0.009
3, n = 13, 8 questions	0.190	−0.470
4, n = 25, 5 questions	−0.360	−0.148
5, n = 20, 6 questions	−0.305	−0.441
6, n = 14, 8 questions	−0.367	−0.332

A negative correlation indicates that more experienced and better-credentialed experts were closer to the truth. A positive correlation indicates the converse. None of the correlations were statistically significant (at α = 0.05, two-tailed).

Figure 1 also shows that most people's self-assessments are lower than the assessments provided by their peers. That is, people tended to score themselves slightly lower than other people scored them, on the 11-point scale, perhaps reflecting innate modesty on the part of most participants, or an innate inflation of other peoples' expertise. Despite this overall bias in scaling, the relative positions of people on the self-assessment and peer assessment axes were remarkably consistent, reflected in the strength of these correlations (Table 2).

Levels of modesty declined as status increased. That is, older, more experienced and better-credentialed people tended to place themselves slightly closer to the parity line than did younger and less experienced people (Figure 1).

**Figure 2:**
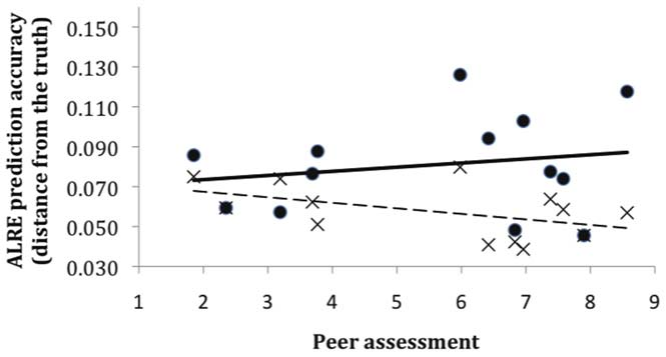
Peer assessment of expert knowledge versus actual performance for the participants in Workshop 3. Prediction accuracy is calculated as ALRE (see text). Small values for prediction accuracy are better. Closed circles and the solid line are estimates from round 1 (*r* = 0.19). Crosses and the dashed line are estimates from round 2 (*r* = −0.47). Estimates closer to the x-axis indicate the answers are closer to the truth.

Despite beliefs in expert performance, the relationships between status (as reflected in the peer assessments) and actual performance on the questions were consistently weak, ranging from −0.39 to 0.22 on the initial (first round) predictions, and from −0.47 to 0.12 on the final (second round) predictions (Table 3). Individual average prediction accuracies improved after people had the opportunity to discuss the context and meaning of questions, and to see the results of the first predictions from the other experts. For example, this is reflected in the round 2 (dashed) line in Figure 2 being below the round 1 (solid) line.

**Figure 3:**
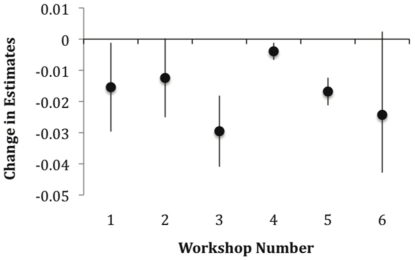
The group average improvement in accuracy (ALRE) following discussion. Change in estimates records distance from the truth, so that more strongly negative values improved more. The dots are the improvements in averages of best guesses. The error bars are 95% confidence intervals.

In the example in Figure 2, before discussion, reputation was weakly positively correlated with improved performance (*r* = 0.19). After discussion, the correlation was the reversed (*r* = −0.47). This result hinged on several of the most experienced participants who improved substantially, following clarification and consideration of other opinions. This example of overall improvement in performance following discussion emerged in all the workshops (Figure 3), where the average accuracy of expert judgements improved substantially following discussion in all cases.

**Figure 4:**
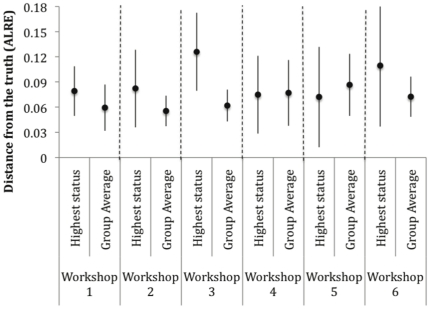
Accuracy of group means compared to highly regarded experts. Mean and 95% confidence intervals of standardized distance from the truth (ALRE) for the most highly regarded individual in each workshop prior to discussion (‘Highest status’), and the workshop group average following discussion (‘Group Average’).

The average estimates performed about as well as, or better than, the best regarded person in each group (Figure 4), although in workshop 5, the best regarded person was the second best performing individual and slightly outperformed the group average. More importantly, the person with the highest status often performs poorly, relative to the post-discussion group average.

## Discussion

These results support three important conclusions. Firstly, they confirm one prediction of the *social expectation hypothesis*, that peer assessments of expert status would be strongly associated with qualifications, track record and experience. Not only does society expect experts to perform better, but experts themselves believe they will perform better. These expectations arise because people believe skill is determined by these factors.

Secondly, in contrast to the *social expectation hypothesis*, qualifications, track records and experience often are poor guides to the performance of scientific experts. Even though each group contained at least one person who was consistently relatively accurate, the results show clearly that peer status cannot be used to identify such people. The only way to identify them is to test participants.

Thirdly, if experts are given the opportunity to listen to one another, assess other judgements, and cross examine reasoning and data within a structured process, their average performance improves substantially. Additionally, the averages of a group's independent best guesses following discussion generally perform at least as well as, and often much better than the estimates of the best-regarded person in the group [20].

These three results were consistent when experts were averaged over a set of questions, in all the workshops conducted here. We speculate that they are general properties of technical expertise.

Others [8], [18], [19] have suggested testing experts routinely and using these data to weight their judgments about unknowns. While our results support the general notion of testing and weighting experts, the prospect of doing this raises challenging questions. Who sets and administers the tests? Which elements of expertise should the tests examine? Where do the data come from to validate the answers? How many test questions are required to validate an individual's expertise? How does one overcome the reluctance of experts to be tested?

The analyses here provide some answers. Regarding setting tests, the people interested in gathering an accurate set of facts for an assessment have a mandate to conduct such tests and a responsibility to eliminate as many arbitrary and misleading sources of uncertainty as possible. Regarding the scope of the questions, they should cover the kinds of questions that the experts will be required to answer to finalize a risk analysis or to make the decision at hand. Regarding sources of potential questions, they include hypotheticals, prepublication data, data from previous case studies that other experts have collected, and data from other locations or jurisdictions. Regarding reluctance, only one person in 124 participants in these workshops refused to make their estimates available for discussion and inclusion in this study. It remains to be seen whether the same responses emerge in other cultures, disciplines and contexts.

These results reinforce earlier findings that if experts have the opportunity to enhance their judgment ability through learning, their performance generally improves [18], [19], [21], [22]. Here, the experts learned from one another and improved their estimates rapidly. These earlier studies have shown that practice and experience alone do not necessarily remove biases. Learning from private feedback is a slow process requiring many iterations. This study shows that learning from peers in a facilitated and a structured environment can accelerate judgment improvement.

In summary traditional measures of expertise such as publication record and years of experience are unreliable predictors of accuracy during elicitation exercises. We therefore recommend a formal and transparent process for the definition and selection of expert panels, which considers other measures of domain knowledge. We also recommend the adoption of new professional standards that employ structured elicitation methods and testing and feedback of expert judgments. It is anticipated that these will improve the performance of both experts and elicitation methods over time.

## Supporting Information

Supporting Information S1 **The information outlines two example questions that represent the style of the test questions used in the six workshops; one example question is for weed ecologists and the other for health epidemiologists.**
(DOC)
