# Supplementary material for: Expert Status and Performance
Source: PLoS One. 2011 Jul 29;6(7):e22998. doi: 10.1371/journal.pone.0022998 (PMC3146531; doi:10.1371/journal.pone.0022998)
Supplement: Supporting Information S1 — The information outlines two example questions that represent the style of the test questions used in the six workshops; one example question is for weed ecologists and the other for health epidemiologists. (DOC) [file pone.0022998.s001.doc]

**Example questions**

*For park managers:*

In 2008, a park manager reported on the extent of Boneseed (*Chrysanthemoides monilifera* subsp. *monilifera*) infestation in the You Yangs National Park in Victoria. It is thought to have been planted for erosion control in the 1940s. By 1963, the district forester reported that ‘moderate to heavy’ infestations of the weed covered 650 ha of the 2000 ha park. **How many of the 2000 ha hectares of the Park did the manager report it covered in 2008?**

*For epidemiologists:*

Q fever, a vaccine preventable bacterial disease, is a hazard for people in the meat and livestock industries. It is spread by inhaling droplets or dust contaminated by infected animals’ urine, milk, faeces or slaughter offal. From 1991 to 1993 the vaccine was used in a few abattoirs. After 1993, vaccination covered large abattoirs in most states. From 2001, farming families and employees in the livestock industry were vaccinated. The average number of cases reported per year in Australia from 1991 to 1993 was 651. **What was the average number of cases reported per year in Australia from 2001 to 2006?**
